# Consensus clinical management guidelines for acid sphingomyelinase deficiency (Niemann–Pick disease types A, B and A/B)

**DOI:** 10.1186/s13023-023-02686-6

**Published:** 2023-04-17

**Authors:** Tarekegn Geberhiwot, Melissa Wasserstein, Subadra Wanninayake, Shaun Christopher Bolton, Andrea Dardis, Anna Lehman, Olivier Lidove, Charlotte Dawson, Roberto Giugliani, Jackie Imrie, Justin Hopkin, James Green, Daniel de Vicente Corbeira, Shyam Madathil, Eugen Mengel, Fatih Ezgü, Magali Pettazzoni, Barbara Sjouke, Carla Hollak, Marie T. Vanier, Margaret McGovern, Edward Schuchman

**Affiliations:** 1 University Hospital Birmingham NHS Foundation Trust, Birmingham, UK; 2 Institute of Metabolism and System Research, University of Birmingham, Birmingham, UK; 3 Children’s Hospital at Montefiore, Albert Einstein College of Medicine, Bronx, NY USA; 4 Regional Coordinator Centre for Rare Disease, AMC Hospital of Udine, Udine, Italy; 5 Department of Medical Genetics, University of British Columbia, Vancouver, BC V6T 1Z2 Canada; 6 Department of Internal Medicine, Hôpital de La Croix Saint Simon, Paris, France; 7 BioDiscovery and DR BRASIL Research Group, HCPA, Department of Genetics and PPGBM, UFRGS, INAGEMP, DASA, and Casa Dos Raros, Porto Alegre, Brazil; 8 International Niemann-Pick Disease Registry, Newcastle, UK; 9 National Niemann-Pick Disease Foundation, Fort Atkinson, WI USA; 10 ASMD España, Madrid, Spain; 11 Department of Respiratory Medicine, University Hospital Birmingham NHS Foundation Trust, Queen Elizabeth Hospital, Birmingham, UK; 12 Institute of Clinical Science in LSD, SphinCS, Hochheim, Germany; 13 Division of Pediatric Metabolism and Division of Pediatric Genetics, Department of Pediatrics, Gazi University Faculty of Medicine, 06560 Ankara, Turkey; 14 Biochemistry and Molecular Biology and Reference Center for Inherited Metabolic Disorders, Hospices Civils de Lyon, 59 Boulevard Pinel, 69677 Bron Cedex, France; 15 Department of Endocrinology and Metabolism, Amsterdam University Medical Centers, Academic Medical Center, University of Amsterdam, F5-169, P.O. Box 22660, 1100 DD Amsterdam, The Netherlands; 16 INSERM, Hospices Civils de Lyon, Lyon, France; 17 Yale School of Medicine, New Haven, CT USA; 18 Department of Genetics and Genomic Sciences, Icahn School of Medicine at Mount Sinai, 1425 Madison Avenue, Room 14-20A, New York, NY 10029 USA

**Keywords:** Acid sphingomyelinase deficiency, ASMD, Niemann–Pick disease, Niemann–Pick disease-a,b,a/b, Guidelines, Diagnosis, Management

## Abstract

**Background:**

Acid Sphingomyelinase Deficiency (ASMD) is a rare autosomal recessive disorder caused by mutations in the *SMPD1* gene. This rarity contributes to misdiagnosis, delayed diagnosis and barriers to good care. There are no published national or international consensus guidelines for the diagnosis and management of patients with ASMD. For these reasons, we have developed clinical guidelines that defines standard of care for ASMD patients.

**Methods:**

The information contained in these guidelines was obtained through a systematic literature review and the experiences of the authors in their care of patients with ASMD. We adopted the Appraisal of Guidelines for Research and Evaluation (AGREE II) system as method of choice for the guideline development process.

**Results:**

The clinical spectrum of ASMD, although a continuum, varies substantially with subtypes ranging from a fatal infantile neurovisceral disorder to an adult-onset chronic visceral disease. We produced 39 conclusive statements and scored them according to level of evidence, strengths of recommendations and expert opinions. In addition, these guidelines have identified knowledge gaps that must be filled by future research.

**Conclusion:**

These guidelines can inform care providers, care funders, patients and their carers about best clinical practice and leads to a step change in the quality of care for patients with ASMD with or without enzyme replacement therapy (ERT).

## Background

Acid Sphingomyelinase Deficiency (ASMD; alternatively known as Niemann–Pick Disease Types A, B and A/B, OMID# 257,200 and 607,616) is an ultra-rare multisystem genetic disorder caused by pathogenic variants of the *SMPD1* gene. Clinical features, time of onset and disease severity can vary greatly among the subtypes and even within families bearing identical genetic alterations. At the severe end of the spectrum, the disease is rapidly progressive in nature and results in premature death. At the milder end, patients may be oligosymptomatic and a diagnosis can be easily overlooked. The rarity of the disease and the scarcity of expertise contribute to misdiagnosis, delayed diagnosis and barriers to adequate care. This may lead to inadequate or inappropriate care, patients’ and families’ loss of confidence in the healthcare system and disempowerment, even though the diagnosis of ASMD, particularly type B, and to a lesser extent type A/B, is compatible with improved quality of life if a diagnosis is made promptly and appropriate disease modifying and supportive management is instituted. There is a disease modifying enzyme replacement therapy (ERT) which has recently received regulatory approval in many countries, therefore, it has become even more essential to redefine standard operating procedures to improve diagnosis and care of ASMD patients using multi-disciplinary and multi-professional teams of experts. This includes symptomatic supportive therapy, still the mainstay of management in many patients, and definitely in those with type A. The Niemann–Pick Disease (NPD) community, represented by the International Niemann–Pick Disease Alliance (INPDA), with support from the International Niemann–Pick Disease Registry (INPDR), has initiated and sponsored the development of comprehensive disease management guidelines to provide a resource for the multi-disciplinary team, and to support patients and their primary professional caregivers on current diagnosis, treatment, monitoring and outcome measures for patients with ASMD. This document represents a general guideline, which in the opinion of the authors can inform care providers about the needs of patients with ASMD in order to provide equitable and improved care, define the standard of care for ASMD patients, identify knowledge gaps, foster shared care arrangements between expert centres and family physicians, and ultimately to empower patients. The guidelines encompass management of patients suspected or diagnosed with ASMD disease at any age. These guidelines should be of value to: a) specialist centres, hospital-based medical teams and staff involved with the care of ASMD patients, b) family physicians and primary caregivers, c) patients and their families d) healthcare funders and regulatory agencies. It was developed by experts with extensive experience of European, North and South American healthcare systems and populations. However, they might equally be applicable to any country that operates similar healthcare services. It is anticipated that implementation of these guidelines will lead to a step change in the quality of care for patients with ASMD.

## Methods

These guidelines were developed by expert physicians, geneticists, allied healthcare professionals and patient support groups involved in the International Niemann–Pick Disease Alliance (INPDA) (www.inpda.org). INPDA is a global network of not-for-profit organisations, supporting individuals affected by Niemann–Pick diseases. In addition, the International Niemann–Pick Disease Registry (INPDR) (www.inpdr.org), a subsidiary of INPDA which has developed a global NPD disease registry, provided support in the development of these guidelines. One of the goals of the INPDA and the INPDR is to support patients and care givers to provide equitable care to Niemann–Pick disease patients by standardizing the quality of care all patients receive.

The Guidelines Development Group (GDG) consisted of expert representatives from a range of professional groups including paediatric and adult metabolic specialists, geneticists, neurologists, hepatologists, pulmonologists, epidemiologists, clinical biochemists, specialist nurses and patient support group representatives. The GDG committee at their first meeting agreed upon the remit of the guidelines and selected a list of guidelines topics for development.

A systematic literature review on ASMD in the last 20 years until December 2021 was carried out using Medline, Embase and the Cochrane Library. The following search-string was used for PubMed, with appropriate modifications for the other two databases: (Acid Sphingomyelinase Deficiency[Text Word]) OR (Niemann–Pick B[Text Word]) OR (Niemann–Pick A[Text Word]). Relevant papers which were previously published and considered by the GDG members as important were included. Searches were limited to English language publications only. The initial search identified 720 reference abstracts, of which 195 were accepted as relevant after the first screen. References related to a single topic (i.e., Epidemiology, Genetics, Pathophysiology, Clinical Diagnosis, Laboratory, Imaging, Therapy, Recommendations) were pulled together and the GDG was divided into subgroups aimed to critically appraise references devoted to a specific topic. More recent papers related to therapeutic options were added at a later stage. The committee met virtually twice (February 2022 and June 2022), face to face once (August 2022) and corresponded by email on a regular basis throughout the duration of the guideline development process. Additionally, an in-person meeting to consult with patients regarding guideline contents took place (August 2022). During the first meeting, the GDG adopted the second version of the Appraisal of Guidelines for Research & Evaluation (AGREE II) system as methodological approach in order to meet the guidelines development standards outlined in the AGREE II system: however, our guidelines didn’t completely meet 5 of the 23 items outlined in the AGREE II system, and we haven’t calculated quality scores for all appraisal items [1].

Relevant papers were evaluated by members of the GDG before the evidence was considered. Sub-group leaders individually assessed the selected literature and wrote a short document containing key questions to be addressed in the topic and drafted key statements and further explanation describing the study findings and related recommendations. All GDG members discussed the draft documents at the virtual and face to face meetings. Evidence levels were classified in accordance with the Grading of Recommendations, Assessment, Development and Evaluations (GRADE) methodology and recommendations were graded from A to C (Table 1). In addition, for the adoption of recommendations, we structured a panel of experts that represented a group of specialists caring for ASMD patients and used the Delphi method for the development of the guidelines. In total, 24 international experts participated, and after the first round of Delphi consensus 5 statements required substantial revision and the expert opinion expressed in the guidelines were based on the revised statements.

**Table 1:** Evidence levels and strength of recommendations

Item	Definition
*Level of evidence*	
A. High quality evidence	Further research is unlikely to change our confidence in the estimate of effect. Consistent evidence from Randomised Controlled Trials (RCTs) without important limitations or exceptionally strong evidence from observational studies
B. Moderate-quality evidence	Further research is likely to have an important impact on our confidence in the estimate of effect and may change the estimate. Evidence from RCTs with important limitations (inconsistent results, methodologic flaws, indirect or imprecise), or very strong evidence from observational studies
C. Low-quality evidence	Further research is very likely to have an important impact on our confidence in the estimate of effect and is likely to change the estimate. Evidence for at least one critical outcome from observational studies, case series, or from RCTs with serious flaws, or indirect evidence, or expert’s consensus
*Strength of recommendation*	
1. Strong recommendation	Recommendation can apply to most patients in most circumstances
2. Weak recommendation	The best course of action may differ depending on circumstances or patient or society values. Other alternatives may be equally reasonable

The guidelines will be published in an open access journal and will be promoted by the INPDA and INPDR to ensure global reach. These guidelines will be reviewed every 3–5 years to reflect any new data pertaining to future research findings, new therapies and the development of diagnostic methods. The development of these guidelines was made without external financial support from industries involved in the manufacturing of therapies for ASMD. Competing interests of members of the guideline development group have been recorded in writing and addressed.

Developing treatment guidelines in an objective and scientific manner for a rare disease is challenging owing to the relative lack of randomized controlled trials (RCT). We have attempted to apply all the AGREE II domains in our guidelines development. However, the AGREE II methodology was developed for common disorders, where there is wealth of evidence in a form of RCTs. Despite our best effort, we found it difficult to apply AGREE II in full for an ultra-rare disorder. We have therefore created guidelines using the best available data and expert opinion.

## Results and discussion

### Definition, epidemiology and pathophysiology

ASMD is a pan-ethnic, ultra-rare, multisystemic, mostly progressive and potentially life limiting metabolic disorder with age of onset varying from first day/months of life to adulthood. ASMD is due to the deficient activity of the enzyme acid sphingomyelinase (ASM). ASM is a lysosomal lipid hydrolase required to degrade the sphingolipid, sphingomyelin, into ceramide and phosphocholine. A deficiency of this enzyme results in sphingomyelin accumulation, representing the underlying pathologic defect.

ASMD is inherited as an autosomal recessive trait that results from the reduced activity of acid sphingomyelinase due to loss-of-function variants in *SMPD1*, the gene encoding ASM [2]. To date, over 250 *SMPD1* variants have been described in ASMD patients, that result in a wide range of clinical phenotypic severity [3, 4]. Carrier individuals who inherit only one pathogenic *SMPD1* allele will be clinically normal. If two carrier individuals have children, each offspring has a 1:4 chance of being affected. There is some evidence indicating that *SMPD1* may be paternally imprinted (preferentially expressed from the maternal chromosome), but it is not known if this has any impact on the ASMD phenotype [5]. The first infant with this disease was described in 1914 by the German paediatrician, Albert Niemann [6]. More such patients were reported, and detailed pathological studies were conducted in 1922–1927 by Ludwig Pick [7]. These infants followed a rapidly neurodegenerative course leading to death within 3 years of age. In 1946, Pfändler and Dusendschon described 2 adult brothers with similar pathologic findings, but distinguishable from Niemann’s patients by a later onset of disease symptoms and the lack of central nervous system (CNS) manifestations [8]. Over the years, the disease became known as “Niemann–Pick Disease” (NPD). In 1958, Crocker and Farber published a review of 18 cases of NPD, showing that there was a wide variability in age of onset and clinical expression, and in the level of sphingomyelin storage in tissues [9]. This led Alan Crocker (1961) to delineate these patients into four subtypes (types A-D). Type A (corresponding to the original patients described by Niemann and Pick) was characterized by severe, early CNS deterioration and visceral and cerebral sphingomyelin storage. Type B showed a chronic course with marked visceral involvement and visceral sphingomyelin storage, but with a sparing of the nervous system. Patients with Types C and D (the latter corresponding to patients from Nova Scotia) were characterized by a subacute nervous system involvement with a moderate and slower course, as well as milder visceral storage. We now know that the latter patients have a different disease caused by mutations in two genes involved in cholesterol cellular trafficking, *NPC1* or *NPC2* [10–12]. In 1966, Brady and associates demonstrated a severe deficiency in acid (lysosomal) sphingomyelinase activity in tissues from patients with type A [13], a finding soon extended to type B. Intermediate phenotypes were subsequently described in patients with ASM deficiency, and these became known as Type A/B NPD [14]. The next step was the identification of the gene encoding acid sphingomyelinase, *SMPD1*, together with that of pathogenic variants in patients, confirming that Niemann–Pick disease types A and B were allelic disorders [2]. The disease is now referred to as ASMD to better describe these patients and distinguish them from those with Type C NPD [15]. The original Type A patients are classified as the **infantile neurovisceral type**; Type B patients are referred to as the **chronic visceral type**, and patients with Type A/B are referred to as the **chronic neurovisceral type**.

#### How common is ASMD deficiency?

*Statement 1*: ASMD is a pan-ethnic ultra-rare, autosomal recessive metabolic disorder, with an estimated global prevalence of ~ 1:100,000–1,000,000 births. There is a higher frequency of some specific SMPD1 pathogenic variants in certain ethnic populations with disease incidence as high as ~ 1:40,000.

- Strength of recommendation: 1
- Level of evidence: B
- Experts’ opinion: completely agree (69%), mostly agree (25%), partially agree (6%), mostly disagree (0%) and completely disagree (0%).

The most extensive genetic screening for ASMD has been performed in the Ashkenazi Jewish population, where the carrier frequency is estimated to be between ~ 1:100 and 1:200 based on screening for the three “Type A” NPD mutations common in this population (predicting a disease prevalence between ~ 1:40,000 and 1:200,000) [16, 17]. Several other studies have estimated carrier or disease frequencies in specific populations, and the results are highly variable. In part, this may be due to founder effects and the incidence of consanguinity. In addition, most of these estimates are based on data from lysosomal storage disease testing laboratories rather than from population-based screening [18–21]. More accurate determination of the true disease incidence must await additional screening data in different populations.

Historically, ASMD is challenging to recognize, likely leading to under diagnosis. In part, this is due to limited awareness within the medical community, the wide-ranging phenotype, and overlap with other, more common conditions. In addition, the number of laboratories offering testing for ASMD activity has been limited. It is currently estimated that several thousand ASMD patients may be living worldwide based on the number of diagnosed cases and limited information on the disease incidence from sources such as Orphanet, which cites a prevalence of 1–9/1,000,000 in Europe. ASMD is probably underestimated and it is unknown how many cases remain undiagnosed and the true incidence of the disease in various populations remains to be elucidated.

#### Pathophysiology

ASMD is a complex lysosomal lipid storage disease that leads to cellular dysfunction in multiple organs. The exact pathophysiology of ASMD is insufficiently understood. Since the disease initiates in lysosomes, a number of abnormalities may be directly linked to the dysfunction of these organelles, including defects in endocytosis/exocytosis, autophagy, and macromolecule turnover. The initial accumulation of lysosomal sphingomyelin leads to the accumulation of other lipids in lysosomes, the most prominent of which is cholesterol [22–24]. Other lipids derived from sphingomyelin, such as ceramide and molecules that derive from it (e.g., sphingosine), or lysosphingomyelin (sphingosylphosphorylcholine), as well as glycosphingolipids and bis (monoacylglycero) phosphate, also accumulate [24]. Over time, as lipids recycle to the cell membrane, these lipids distribute to other compartments, causing a wide range of secondary cellular abnormalities. These may include plasma membrane signaling abnormalities that trigger inflammation and apoptosis, respiration abnormalities due to mitochondrial defects, and abnormalities in nuclear transport.

Other than mature erythrocytes, all cells have lysosomes. As such, all cells will be impacted by the deficiency of ASM. However, cells of the monocyte/macrophage system are actively involved in phagocytosis and have abundant lysosomes, and are therefore impacted the most in ASMD. Typical histologic images of reticuloendothelial organs, including the liver and spleen, reveal large numbers of lipid-laden macrophages infiltrating throughout the tissues [7]. The same is true in the lung, where these cells may be found primarily in alveoli as well as in interstitial tissue. Other organs significantly impacted in ASMD include the heart, skeletal system, lymphatic and haematopoietic systems, and in some patients, the CNS [25–27]. In these latter patients, storage can be found in glia cells as well as neurons.

## Clinical signs and symptoms

### How is ASMD classified?

*Statement 2*: The clinical manifestation and life expectancy of ASMD patients varies substantially according to the subtypes. ASMD broadly can be divided into infantile neurovisceral ASMD A; chronic neurovisceral ASMD A/B and chronic visceral ASMD B. While the grouping of patients into designated subtypes is helpful, ASMD patients present with a wide range of phenotypes along a continuum disease spectrum.

- Strength of recommendation: 1
- Level of evidence: B
- Experts’ opinion: completely agree (100%), mostly agree (0%), partially agree (0%), mostly disagree (0%) and completely disagree (0%).

Historically, patients with ASMD presenting with severe infantile neurovisceral disease have been categorized as NPD type A, whereas those who have primarily visceral disease have been classified as NPD type B. However, some patients may have protracted neurovisceral disease [17, 28–30] and are described in the literature as variant NPD type B, NPD type A/B, or intermediate NPD [15]. It has increasingly become apparent that many patients may not be easily classified, and even within subtypes there is substantial variability. For example, within the chronic, visceral disease subtype (NPD type B), there are patients diagnosed as young children with severe liver, pulmonary and/or splenic disease, and other patients who are diagnosed as adults with much milder disease, without clinical progression over years [30–32]. Within the chronic neurovisceral phenotype some patients experience developmental delay, ataxia, and/or progressive neurologic deterioration, while others may exhibit learning or behavioral abnormalities without any evidence of progression [33]. Thus, the designated nosology is a useful tool, but the disease exhibits a continuum of phenotypes. An alternate classification system and how it relates to the traditional designations is shown in Table 2.

**Table 2:** Classification of patients with ASMD

	Infantile neurovisceral (ASMD type A)	Chronic neurovisceral (intermediate ASMD, ASMD A/B, variant ASMD B)	Chronic visceral (ASMD type B)
Phenotype	Infantile onset of severe visceral involvement and neurodegeneration with progressive psychomotor deterioration	Visceral features of NPD B as well as neurologic findings including ataxia, variable degrees of developmental delay and peripheral neuropathy	Chronic progressive multi-system or oligosymptomatic, stable disease with no or little neurologic involvement
Natural History	Relentless progression of neurologic and visceral disease and death typically by 3 years of age	Wide spectrum of disease manifestations and severity, with slow progression Patients live past early childhood and sometimes into adulthood	Wide spectrum of disease manifestations and severity. Sometimes oligosymptomatic. Survival usually extends well into adulthood and may even be normal. Patient may remain stable for years

### What is the clinical presentation in Infantile Neurovisceral ASMD type A?

*Statement 3*: Early development of hepatosplenomegaly in infancy with initial achievement of developmental milestones until about 6 months of age followed by progressive neurological deterioration starting in the first year of life, especially if associated with a macular cherry red spot, should raise the suspicion of a lysosomal storage disorder including ASMD type A.

- Strength of recommendation: 1
- Level of evidence: B
- Experts’ opinion: completely agree (73%), mostly agree (27%), partially agree (0%), mostly disagree (0%) and completely disagree (0%).

ASMD type A is the most severe form of ASMD and has a relatively uniform natural history characterized by severe progressive neurodegenerative manifestation in the first year of life with hepatosplenomegaly, development arrest and hypotonia and death typically by 3 years of age [29, 30]. Most infants present with hepatosplenomegaly at 2–4 months of age [29] followed by onset of neurologic symptoms at a median age of 7 months, developmental arrest by 10 months of age and then rapidly progressing neurodegeneration with deterioration of behavioral, language and gross and fine motor skills. Patients show progressive hypotonia with loss of deep tendon reflexes, whereas cranial nerve function remains largely intact. Macular cherry-red spots are detectable in most infants by 12 months. These infants suffer from failure to thrive due to insufficient intake of calories resulting from worsening hypotonia, weakened suck and gastrointestinal symptoms. All infants develop progressive restrictive pattern of respiratory symptoms due to the accumulation of Niemann–Pick cells in the alveolar septa, bronchial walls, and pleura with frequent respiratory infections secondary to aspiration, and most die of respiratory failure. Massive hepatosplenomegaly is typically present, and most infants have significant hepatic dysfunction or liver failure. Abnormal laboratory values include elevated liver enzymes, low HDL cholesterol and progressive decreases in hemoglobin values and platelet counts. (Table 3).

**Table 3:** Classification of patients with ASMD

	Acute neurovisceral, ASMD type-A	Chronic neurovisceral, ASMD type-A/B	Chronic visceral; ASMD type-B
Hepatosplenomegaly	+	+	+
Proatherogenic lipid profile	+	+	+
Delayed growth and puberty	N/A	+	+
Thrombocytopenia	+	+	+
Interstitial lung disease	+	+	+
Skeletal involvement	+	+	+
Liver disease	+	+	+
Cherry red macula	+	Some patients	Some patients
Hypotonia	+	Some patient	Absent
Neurodegeneration	Rapidly progressive	Slowly progressive	Absent

### What is the clinical presentation in Chronic Visceral ASMD type B?

*Statement 4*: The onset of ASMD type B is variable, from childhood to adulthood. Patients with ASMD type B have extensive phenotypic heterogeneity in disease manifestations, from asymptomatic to polysymptomatic. Progression of disease can vary considerably and stability can also be seen in attenuated older patients. The most common symptoms at initial presentation are splenomegaly and hepatomegaly. Common historical symptoms and complaints may include bleeding, shortness of breath, pulmonary involvement, joint and/or limb pain, bruising, headaches, diarrhea and bone fractures. Interstitial lung disease, abnormal blood counts and/or atherogenic lipid profile can be seen.

- Strength of recommendation: 1
- Level of evidence: B
- Experts’ opinion: completely agree (63%), mostly agree (25%), partially agree (12%), mostly disagree (0%) and completely disagree (0%).

Being a rare disease and having variable phenotypic manifestations lead to misdiagnosis of ASMD, especially in the later onset group (type B). The systemic involvement in later onset ASMD (types A/B and B) can present anytime from early childhood to adulthood and sometimes requires a high index of suspicion to make a diagnosis.

### What are the neurologic features in patients with Chronic Neurovisceral variant (ASMD A/B or intermediate ASMD)?

*Statement 5*: Patients with variant forms of ASMD have an intermediate phenotype between A and B, with important somatic manifestations outlined in statement 6 and a slowly progressive neurological disease ranging from mild hypotonia or hyporeflexia to severe progressive neurologic abnormalities such as ataxia, spasticity and cognitive decline. In these patients, the onset of neurologic disease is later in life than in patients with ASMD type A and is more indolent.

- Strength of recommendation: 1
- Level of evidence: B
- Experts’ opinion: completely agree (56%), mostly agree (44%), partially agree (0%), mostly disagree (0%) and completely disagree (0%).

Patients with variant forms of ASMD have been described in several reports [14, 25, 30, 34–39]*.* In a prospective study of 64 patients with ASMD type B who underwent detailed neurologic and ophthalmologic examinations, 19 (30%) had neurological abnormalities, suggesting that patients with a phenotype that includes neurological manifestations constitute a significant proportion of ASMD patients [37]. In some cases the onset of neurological and neuropsychiatric symptoms is subtle and insidious. Worsening neurological symptoms and signs after an initial period of normal development would be a clue. Similarly, in a report of 25 Czech and Slovak patients with ASMD who did not demonstrate the classic ASMD type A phenotype, 16 (64%) had neurologic symptoms [14]. In this series, 12 of the 16 patients had the p.Q294K mutation in homoallelic or heteroallelic form, and 10 of those had a protracted neurovisceral phenotype [14]. p.W393G is a variant found in south-east Europe (Sinti and Romanies background); these patients frequently show neuropsychiatric symptoms.

### What are the visceral manifestations of ASMD?

*Statement 6*: Splenomegaly is one of the most common disease manifestations of ASMD and often the first obvious sign of the disease. Another common visceral manifestation is hepatomegaly. Most patients have radiologic evidence of interstitial lung disease.

- Strength of recommendation: 1
- Level of evidence: B
- Experts’ opinion: completely agree (63%), mostly agree (31%), partially agree (6%), mostly disagree (0%) and completely disagree (0%).

Hepatosplenomegaly is one of the commonest manifestations of ASMD and the differential diagnosis would include primary liver disorders including chronic hepatitis B and other infections, lymphoma and other malignancies, other storage disorders like Gaucher disease, lysosomal acid lipase deficiency (LAL-D) and Niemann–Pick type C (NPC) disease. Diagnostic work-up for Gaucher disease, LAL-D and NPC should regularly and simultaneously include ASMD. Splenomegaly can be massive and may be a surrogate marker of disease severity because of its correlation with other disease parameters [40, 41].

Elevation of alanine aminotransferase (ALT), aspartate aminotransferase (AST) and total bilirubin can be seen [41]. A systematic analysis of liver biopsies from adult patients with ASMD type B revealed the presence of liver fibrosis in most patients, some of whom had frank cirrhosis in the absence of any clinical symptoms of liver failure [27]. Patients homozygous for the *SMPD1* p.A359D mutation associated with moderate to severe ASMD type B, have a high incidence of clinically relevant liver disease due to progressive cirrhosis [42]. In addition to respiratory disease, liver failure is a common cause of death in patients with ASMD type B [43].

Most patients [41, 44] have evidence of interstitial lung disease by chest radiography and high-resolution computed tomography. However, there is no strong correlation between radiologic findings, respiratory symptoms and the results of pulmonary function tests [44]. Therefore, imaging studies are not sufficient in the evaluation of pulmonary disease in ASMD type B and must be interpreted in conjunction with functional testing and the clinical status of the patient. Overall, respiratory disease is one of the most common manifestations and a leading cause of death in patients with ASMD type B [38, 43]. The combination of hepatosplenomegaly with interstitial lung disease is a useful pointer to the possibility of a multiorgan disease like ASMD. In adults, sarcoidosis is a common cause of interstitial lung disease with multiorgan involvement, but the breathlessness in sarcoidosis would often be far greater for the same extent of radiological changes than in ASMD. The fibrosis in sarcoidosis is often upper lobe predominant and hepatosplenomegaly if present much less obvious. Pulmonary fibrosis can be the main manifestation of other lung disease such as idiopathic fibrosis and pulmonary alveolar proteinosis with minimal hepatosplenomegaly as an additional feature [30].

Dual X-ray Absorptiometry scans to measure bone mineral content (BMC) and bone mineral density (BMD) [45] demonstrate that paediatric patients have significant decreases in adjusted mean BMC and BMD at the lumbar spine, hip and femoral neck compared with a cohort of healthy age-matched subjects. In addition, adults with ASMD type B may have osteopenia or osteoporosis at one or more sites according to the World Health Organization classification of BMD [45]. Thus, skeletal involvement may be a feature of ASMD.

Most patients also have an atherogenic lipid profile characterized by low HDL cholesterol, high total cholesterol, high triglycerides and high LDL and very low-density lipoprotein cholesterol compared with age- and gender-matched control subjects. In a study of paediatric patients with ASMD, including 10 with type A and 30 with type B, all patients displayed abnormal fasting lipid profiles [46]. Furthermore, electron beam tomography of the coronary arteries performed in 18 NPD type B patients revealed positive calcium scores (range 1.4–34.5) in 10 patients, which in patients < 18 years of age suggests the presence of early atherosclerosis [47]. It has been reported that electrocardiogram abnormalities may be present, although with none specific findings such as sinus bradycardia, left ventricular hypertrophy and conduction abnormalities [47]. Mild valvular heart diseases are reported in a few patients with ASMD [47]. In general, there is no strong evidence of primary involvement of the heart in ASMD, but further studies are warranted.

### What are the haematologic findings in ASMD?

*Statement 7*: Patients with ASMD have a variable degree of thrombocytopenia, leukopenia and anemia.

- Strength of recommendation: 1
- Level of evidence: A
- Experts’ opinion: completely agree (63%), mostly agree (37%), partially agree (0%), mostly disagree (0%) and completely disagree (0%).

Mild thrombocytopenia is the most common haematologic abnormality in ASMD. Bleeding episodes may include recurrent epistaxis sometimes requiring repeated cauterizations. Significant bleeding events that have been reported include subdural haematoma, hematemesis, hemoptysis, hemothorax, excessive bleeding after tonsillectomy and adenoidectomy resulting in a blood transfusion, menorrhagia and uterine bleeding that required a hysterectomy [41]. Anemia and leukopenia also may be present [41]. Bleeding disproportionate to injury or after surgical procedures and easy bruising due to thrombocytopenia are more common than anemia.

### What is the impact of ASMD on growth?

*Statement 8*: Growth restriction is common in children with ASMD. Many patients diagnosed with ASMD in childhood have below average height and weight particularly during adolescence. Typically, height and weight is within normal range by adulthood.

- Strength of recommendation: 1
- Level of evidence: B
- Experts’ opinion: completely agree (50%), mostly agree (50%), partially agree (0%), mostly disagree (0%) and completely disagree (0%).

Growth delay is most pronounced in adolescents, who also may have delayed bone age that corresponds with delayed onset of puberty. However, most adults (≥ 18 years of age) have heights in the low normal range, suggesting that a period of catch-up growth occurs in late adolescence and/or early adulthood [41]. Thus, although short stature is a cause of concern for adolescent patients with ASMD type B, final adult heights appear to approach normal values in most patients.

### What are the impacts of ASMD on Quality of Life?

*Statement 9*: Few studies have collected information about the impact of ASMD on health-related quality of life (QoL) and the psychosocial burden of ASMD. ASMD type A is devastating for the patient and the family.

- Strength of recommendation: 1
- Level of evidence: B
- Experts’ opinion: completely agree (81%), mostly agree (19%), partially agree (0%), mostly disagree (0%) and completely disagree (0%).

ASMD type A has a devastating physical and emotional impact for the affected children and their families. Frequently, infants with ASMD type A have irritability, sleep disturbances and insomnia, prolonged periods of crying and frequent vomiting [29]. The need for constant care for these infants has a profound negative effect on caregivers’ QoL. A single study [41] that assessed QoL in a small number of patients with NPD type B using generic QoL instruments (i.e., the Child Health Questionnaire – Parental Form 50 for paediatric patients [CHQ-PF50] and the Short-Form 36 [SF-36] for adults) suggested diminished QoL associated with physical functioning, mental health, general health perceptions and/or emotional well-being in a subset of patients. The psychosocial impact of the disease has been evaluated in a small number of patients with ASMD type B and identified psychosocial problems due to limited physical activity and social isolation [48, 49]. Overall, there is a paucity of quantitative and qualitative data on QoL and the impact of the disease on patients and families [49].

## Diagnostic investigations

**Fig. 1:**
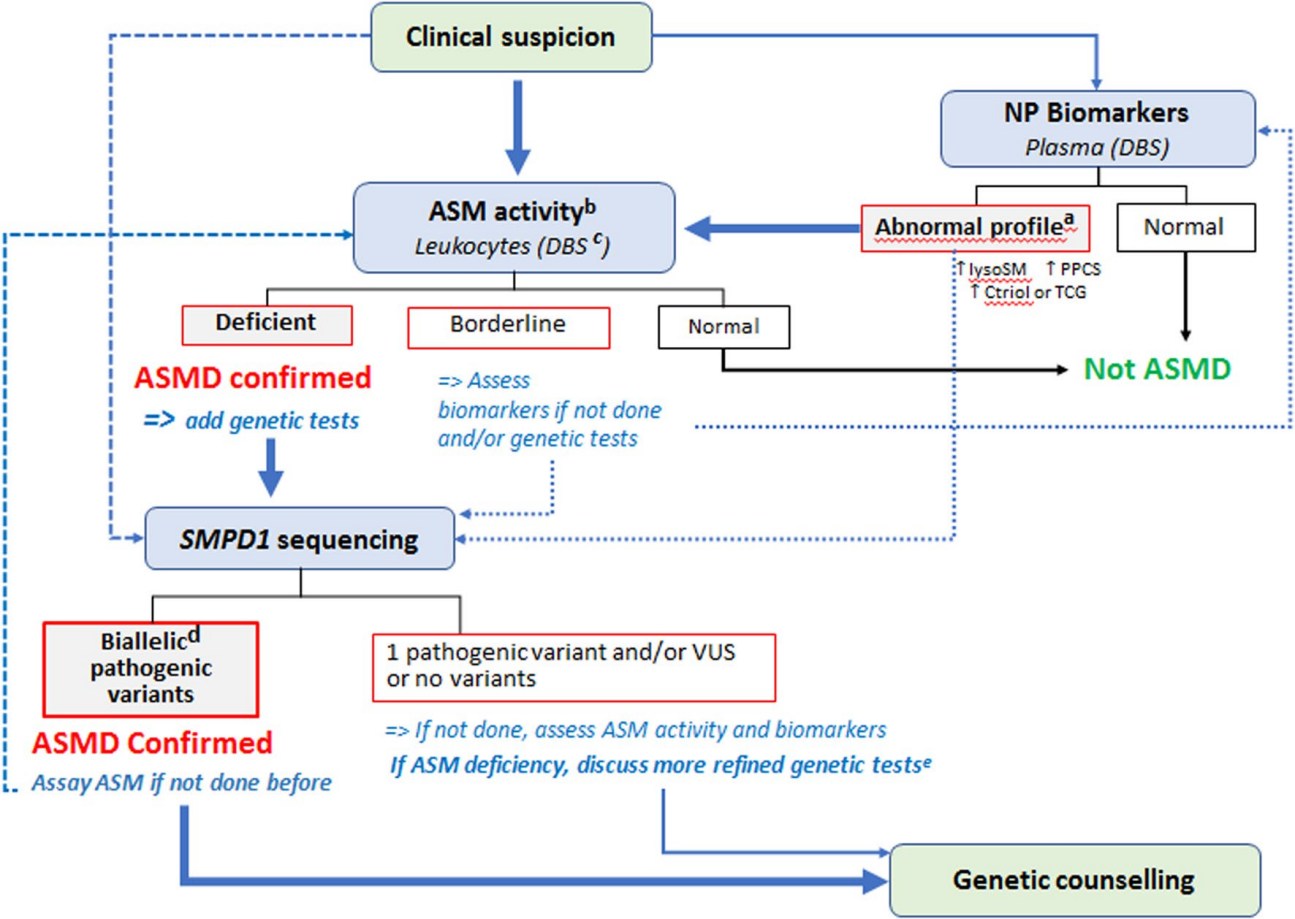
Algorithm for the laboratory diagnosis of ASMD. (**a**) Significant increase of 3β,5α,6β-cholestane-triol (C-triol), 7-ketocholesterol (7-KC), 3β,5α,6β-trihydroxy-cholanoyl-glycine (TCG), lysosphingomyelin (lyso-SM), *N*-palmitoyl-*O*-phosphocholine-serine (PPCS). A difference with an NPC profile is the normal or slightly elevated lyso-SM level in the latter. Other causes of elevated C-triol levels include cerebro tendinous xanthomatosis (CTX) and acid lipase deficiency. (**b**) LC–MS/MS (or radioisotopic) preferred methods (see Statement 13). (**c**) Recommendation for ASM in DBS to be confirmed on leukocytes or by genetic testing. (**d**) Importance of parental study. **(e)** MLPA/RNA analysis. VUS: Variant of uncertain significance

Being a rare disease and having variable phenotypic manifestations may lead to misdiagnosis of ASMD, especially in the later onset group (type B or A/B). ASMD has several laboratory/imaging manifestations; some of them are non-specific but may be detected by routine tests before a specific suspicion is raised. A close look at the combination of results of these tests may lead to the suspicion of ASMD and consequently to the request of a specific diagnostic test (Fig. 1).

### General laboratory tests

BLOOD COUNTS: Patients with ASMD usually present with mild thrombocytopenia, which can be detected in a routine blood count. Anemia may be present as well; also, leucopenia/neutropenia can be present [31].

LIPID PROFILE: An abnormal lipid profile is common in patients with ASMD, the most frequent abnormalities include increased levels of triglycerides and total cholesterol, with low HDL-cholesterol levels [31].

IMAGING: The increased sizes of liver and spleen can be observed incidentally on physical examination or plain radiography, but to measure the volume and to evaluate echogenity it is recommended to perform ultrasonography. More accurate measurements can be obtained with tomography or preferably with MRI. Interstitial lung disease can be suspected from chest X-ray or CT.

### Bone marrow and tissue biopsies

Although neither needed nor recommended for the diagnosis of ASMD, adult patients are often subjected to a bone marrow biopsy due to the suspicion of malignancy in a subject with unexplained splenomegaly. In the bone marrow, ASMD patients will display foamy macrophages (“Niemann–Pick” cells) and/or typical “sea blue histiocytes”. These storage cells are not specific for ASMD. Both types can be present in bone marrow aspirates from patients with NPC and similar foamy macrophages can also be found in other lysosomal storage disorders such as acid lipase deficiency or GM1 gangliosidosis (and also in Tangier disease and some other conditions). Although having a different appearance and staining properties, Gaucher cells may appear similar to less experienced examiners using standard (Giemsa) staining, resulting in potential misdiagnosis.

### Laboratory tests essential to confirm the diagnosis of ASMD

*Statement 10*: Whenever ASMD is suspected, an enzyme assay for ASM activity should be performed. The diagnosis is established by demonstration of deficient or very significantly diminished ASM activity in leucocytes or fibroblasts. Biomarkers can be helpful in combination with enzyme activity measurement to establish the diagnosis especially if enzyme activity measurement is performed in dried blood spots.

- Strength of recommendation: 1
- Level of evidence: A
- Experts’ opinion: completely agree (81%), mostly agree (19%), partially agree (0%), mostly disagree (0%) and completely disagree (0%).

#### Biological sources for assay of acid sphingomyelinase activity

Peripheral blood leukocytes/lymphocytes or dried blood spots (DBS) are the usual biological sources; isolated lymphocytes/monocytes have a specific activity two–threefold higher than total leukocytes. Cultured skin fibroblasts have a much higher level of specific activity than leukocytes (× 40–50) and may be useful in equivocal cases. The use of DBS has become increasingly popular in the diagnosis of lysosomal diseases due to its convenience but it has limitations. Results can be influenced by anemia, leucopenia, and recent transfusions [15]. Pre-analytical conditions for sampling and storage conditions are also important since enzyme activities in DBS are highly sensitive to heat and humidity [50, 51]. Regarding acid sphingomyelinase activity, contrary to most other lysosomal enzymes, the assay had so far been restricted to a very limited number of laboratories, due to its technical complexity. This situation has changed since the recent development of a commercial kit designed for measurements on DBS by tandem mass spectrometry. Consequently, an increasing number of first line diagnoses are currently made on DBS samples. Nonetheless, a diagnosis made by DBS should be confirmed in leucocytes (or fibroblasts), or corroborated with the finding of biallelic pathogenic *SMPD1* variants. Similarly, more false-negative results can occur on DBS than on leukocyte samples and in case of strong clinical suspicion, further investigation of ASM activity in leucocytes or fibroblasts is recommended. In both cases, an elevation of lysosphingomyelin or PPCS biomarkers constitutes useful indicators.

#### Is there an optimal method for determination of acid sphingomyelinase activity?

*Statement 11*: For determination of acid sphingomyelinase activity, the choice of a specific substrate is critical. Use of a short-chain fatty acid sphingomyelin analogue with detection by tandem mass spectrometry (MS/MS) is considered the method of choice.

- Strength of recommendation: 1
- Level of evidence: B
- Experts’ opinion: completely agree (67%), mostly agree (33%), partially agree (0%), mostly disagree (0%) and completely disagree (0%).

#### Evaluation of different methods for assay of ASM activity

Accurate and sensitive methods using natural sphingomyelin radioactively labeled on the choline moiety, historically developed and validated by expert laboratories [52–56], are still excellent. However, due to the need of periodical purification of the substrate and of licensing for use of radiochemicals, this methodological approach is currently on the wane.

A synthetic fluorogenic substrate, 6-hexadecanoylamino-4MU-phosphorylcholine, was proposed as a simpler replacement for radiolabeled sphingomyelin [57]. Patients with some pathogenic *SMPD1* variants such as the p.Q294K [35] were not detected by the original method. This problem could be solved by assessing the extent of inhibition of enzymatic hydrolysis of the artificial substrate by an unlabeled natural substrate, in particular lysosphingomyelin [57]. This substrate, however, has a low sensitivity and provides a lesser discrimination between unaffected and affected subjects than that used in tandem mass spectrometric (MS/MS) assays [58, 59].

Radioisotopic and fluorimetric assays have been superseded by highly sensitive techniques using a short-chain fatty acid sphingomyelin analogue and detection by MS/MS, now considered the method of choice [15], with a good specificity [59] and adaptability to DBS with a high throughput [60]. Multiplex enzyme assays kits using this principle for simultaneous diagnosis of six lysosomal storage diseases are commercialized [61]. They allow, among others, simultaneous testing for ASMD and Gaucher disease, as has recently been recommended [15]. They can also be used for neonatal screening [58, 61–63].

#### Evaluation of different methods for assay of ASM activity

**Statement 12**: Although type B patients often show a slightly higher level of residual activity, the in vitro assay does not reliably distinguish neuronopathic from non-neuronopathic phenotypes.

- Strength of recommendation: 1
- Level of evidence: B
- Experts’ opinion: completely agree (81%), mostly agree (19%), partially agree (0%), mostly disagree (0%) and completely disagree (0%).

Whatever substrate is used, in vitro assays of ASM activity have primarily been designed to optimally demonstrate a deficiency. They do not reflect the true in vivo activity and will not allow a reliable distinction between clinical phenotypes. The in situ loading tests in living fibroblasts [14, 64–66] were more informative, but they are no longer offered by diagnostic laboratories.

### Are there useful plasma biomarkers for diagnosis of ASMD?

*Statement 13*: Several plasma biomarkers show abnormally high levels in ASMD. Despite a lack of specificity for some of them, they can constitute a good first line test before measuring ASM enzyme activity or in combination with enzyme activity measurement. To date, the most specific one is sphingosylphosphorylcholine (SPC) also called lysosphingomyelin (lyso-SM). Its measurement can optimally be combined with that of *N*-palmitoyl-*O*-phosphocholine-serine (PPCS), previously named lysosphingomyelin-509 (lyso-SM509).

- Strength of recommendation: 2
- Level of evidence: B
- Experts’ opinion: completely agree (69%), mostly agree (31%), partially agree (0%), mostly disagree (0%) and completely disagree (0%).

#### Chitotriosidase activity

The activity of chitotriosidase, a human chitinase secreted by macrophages, is strikingly elevated in Gaucher disease [67], and increased to varying degrees in many lysosomal storage diseases (LSDs), as well as in some other disorders in which macrophages are activated. Its assay which only requires simple equipment is widely available. It is therefore still proposed by many laboratories as a first screening test whenever LSD is suspected, in spite of its lack of specificity, and the fact that a number of individuals show a partial or complete deficiency of activity due to common 24-bp duplication in exon 10 of the *CHIT1* gene [68]. In ASMD, elevation is generally modest [69], not constant, and it does not differentiate ASMD from Niemann–Pick type C (NPC), nor from several other diseases with (hepato) splenomegaly.

#### Oxysterol and bile acid derivatives

The oxysterols cholestane-3β,5α,6β-triol (C-triol) and 7-ketocholesterol (7KC), first identified in the search for plasma biomarkers of NPC [70], were found to be also elevated in ASMD [71–73]. But they also show increases in a number of other diseases, including differential diagnoses of ASMD such as acid lipase deficiency or some causes of neonatal cholestasis [74]. The more stable bile acid derivative of C-triol, N-(3β,5α,6β-trihydroxycholan-24-oyl) glycine (TCG), is also increased in both NPC and ASMD [75] [76].

#### Lysosphingomyelin and PPC/lyso-SM509

The most specific plasma biomarker in ASMD to date is sphingosylphosphorylcholine (or lysosphingomyelin, lyso-SM), corresponding to the deacylated form of sphingomyelin, the primary accumulated lipid [32, 77–81]. A marked increase is only observed in ASMD, compared to a small and inconsistent two–threefold increase in NPC [77, 82], and in some metabolic syndrome patients [83]. A pilot study suggests that lyso-SM levels are positively associated to the degree of clinical severity of the patients [84]. Of note, plasma lyso-SM levels have been used and proven useful in follow-up of a recent enzyme replacement therapy trials [85, 86].

*N*-palmitoyl-*O*-phosphocholine-serine (PPCS) initially named lysosphingomyelin-509 [72] is a sensitive biomarker corresponding to a novel class of lipids [87, 88]. It is strikingly elevated in both ASMD and NPC; a modest increase may occur in some other conditions [78, 79]. In ASMD, nominal concentrations of PPCS are much higher than those of Lyso-SM. Until 2019 only the molecular mass (509 Da) of this compound was known (hence its initial name) [72], and not its exact structure. Therefore, in currently published work, lyso-SM509 semi-quantitative measurements have been made with external calibration using a lyso-SM standard for both molecules, with quantification of lyso-SM509 expressed in multiple of median of controls (MoM). Thus far, exact quantifications of PPCS in ASMD patients using homemade standards (in genuine nmol/L) have only been reported from Ory’s and Maekawa’s groups, respectively [89, 90]. Determination of lyso-SM and PPCS is also possible in DBS although less discriminative than in plasma [77, 91, 92].

#### What is the advantage of a multiplex lysosphingolipids and PPCS/lyso-SM509 measurement?

*Statement 14*: Simultaneous measurement of lyso-SM, PPCS and lyso-Gb1 (glucosylsphingosine) can provide good indication for differential diagnosis of ASMD, Gaucher disease, or NPC.

- Strength of recommendation: 1
- Level of evidence: B
- Experts’ opinion: completely agree (50%), mostly agree (50%), partially agree (0%), mostly disagree (0%) and completely disagree (0%).

MS/MS multiplex methods have been developed [78–80, 91], allowing the simultaneous measurement in the same assay of lyso-SM, PPCS, lyso-Gb1 (the best biomarker for Gaucher disease), lyso-Gb3 (biomarker for Fabry disease) and other lyso-glycosphingolipids. Such panels have proven useful in screening of sphingolipidoses and NPC. Striking elevation of lyso-Gb1 allows quick differential diagnosis of Gaucher disease, while a marked elevation of both lyso-SM and PPCS is the signature of ASMD, but not that of NPC.

### Genetic testing

*Statement 15*: Genetic testing of SMPD1 gene should be performed to confirm diagnosis in subjects with ASM activity below normal reference intervals and allows genetic counselling. If SMPD1 sequencing is done before assessing ASM activity and the identified variant for 1 or 2 alleles is not known as pathogenic, demonstration of a deficient ASM activity is mandatory to confirm the diagnosis. Measurement of biomarkers can also be helpful.

- Strength of recommendation: 1
- Level of evidence: B
- Experts’ opinion: completely agree (81%), mostly agree (19%), partially agree (0%), mostly disagree (0%) and completely disagree (0%).

ASMD is caused by biallelic pathogenic variants in the *SMPD1* gene [2, 93]. Molecular analysis of *SMPD1* is highly recommended to support the diagnosis of ASMD; it is the only reliable method for carrier identification within family members, and it is the preferred method for prenatal diagnosis. In addition, it might be useful to establish genotype/phenotype correlations.

The *SMPD1* gene (GenBank#NC_000011.10) spans ~ 5 kb of chromosome 11 (11p15.1–11p15.4) and consists of six exons [2, 93–95]. Although two in-frame translation initiation ATG codons located 32 codons apart from each other have been described, *SMPD1* variants are described according to current mutation nomenclature guidelines (http://www.hgvs.org/mutnomen) [96], ascribing the A of the first ATG translational initiation codon as nucleotide + 1 (GeneBank accession number, NM_000543.4). However, two different cDNA reference sequences have been used to number the *SMPD1* variants (GeneBank accession number, NM_000543.4 and M81780.1) that differed in the length of a highly polymorphic hexanucleotide sequence, hexanucleotide GCTGGC (p.L37_A38), located in exon 1 within the region encoding the signal peptide. Therefore, reports of genetic tests should always include the transcript reference number considered to numbered variants. In this manuscript sequence reference NM_000543.4 is used.

To date, more than 346 variants have been described throughout the whole *SMPD1* gene; among them 295 have been classified as disease associated variants in HGMD Professional 2022.1. All kinds of point mutations have been reported in ASMD patients, including missense, nonsense, frameshift, indels and intronic variants, while only 3 large alterations (gross deletions, deletions/duplications, repeat variations) have been identified so far.

Considering this mutational spectrum, sequencing analysis of the *SMPD1* exons and the intronic flanking regions should be performed as a primary genetic test to confirm/support the diagnosis of ASMD. Sanger or parallel next generation sequencing (NGS) could be used to analyze *SMPD1* as a single gene or as part of a gene panel, respectively [97, 98]. Segregation of alleles by identifying variants in parents should be determined.

The presence of homozygous pathogenic variants not confirmed in parents, as well as the absence of pathogenic variants (in one or both allele) after sequencing should always be questioned and the presence of possible undetected copy number variations (CNV) or deep intronic pathogenic variants should be investigated.

Newly identified variants should be classified following current ACMG/AMP criteria and in case of identification of variants of uncertain significance (VUS), pathogenicity should be tested by functional analysis [99].

#### Genotype/phenotype correlation

The spectrum of reported ASMD associated *SMPD1* variants is extremely heterogeneous, and their frequency and distribution vary among different populations and ethnic groups [14, 30, 32, 42, 100–108], Most mutations have been found in single families and in compound heterozygosity. Therefore, it is quite difficult to correlate the genotype with the phenotype. However, some assumptions can be made based on functional analysis of single mutants and for recurrent mutations found in homozygosity [109].

While the presence of nonsense variants, large deletions or variants leading to a reading frameshift in both alleles is associated with the severe neurovisceral phenotype, a correlation with a specific clinical phenotype is more difficult to establish for pathogenic missense variants. However, the current evidence indicates that patients carrying at least one *SMPD1* pathogenic allele leading to the synthesis of a partially active ASM protein present with the visceral non-neurological phenotype.

Some exemptions apply to this general consideration. Indeed, although the p.W32X mutation, commonly found in Italian patients, would be expected to lead to complete absence of expression of an active protein, it has been nonetheless associated with the non-neurological phenotype. This data suggests that in vivo, when the first ATG is present but unable to produce a canonical transcript, the second initiation codon (ATG33) may be used resulting in the synthesis of a protein missing the first 32 residues of the predicted signal peptide but still partially active [110].

A similar hypothesis can be proposed for the p.R3AfsX76 mutation predicted to introduce a premature stop codon. In fact, even in the absence of in vitro evidence, this mutation is frequently found among ASMD type B Chinese patients even in homozygous status [106].

Regarding recurrent variants, the most frequently reported mutation worldwide is a 3-base deletion, leading to the deletion of arginine at position 610 of the protein (p.R610del) [108]. This variant is associated with high residual ASM activity [102–104, 108, 111] and has been always identified in patients with the non-neuronopathic phenotype [103, 108, 112]. However, a wide range of disease severity has been observed in homoallelic patients. This variant is highly prevalent (> 90% of alleles) in patients originating from Maghreb (Tunisia, Algeria, Morocco) and also frequent in patients of Spanish and French descent [103, 108, 112], rarer in Italy [102] or Poland [32], and has not been reported from China.

The most frequent missense variants identified in Ashkenazim, p.L304P and p.R498L, have been associated to the severe neuronopathic phenotype [113–115]; together with the frameshift mutation p.F333SfsX52 [116], they account for 90% of the alleles found in ASMD patients affected by the infantile neurovisceral phenotype in this population.

The p.Q294K variant has been found in patients from different populations but it is highly frequent in patients from Czech and Slovak heritage [14]. Its presence, both in homozygosity or compound heterozygosity, has been associated with the intermediate phenotype [14].

The missense variant p.W393G is frequent in patients originating from the Western Balkan region and present in 100% of the alleles in those belonging to the Sinti / Roma population [36] It was first described in a family from Serbian origin [34]; these patients were classified as affected by an intermediate clinical phenotype due to the presence of a macular halo and a low in situ ASM activity [34]. However, they were all neurologically normal and we now know that macular halo can occur in typical non-neurological type B patients. Nevertheless, a variability of the phenotype has been described within a genetically homogeneous cohort of 20 Gypsy patients with this mutation, since some of them developed signs of nervous system involvement (cognitive impairment, psychiatric troubles, ataxia, peripheral neuropathy) [36].

## Management

ASMD is not yet curable, but it is a treatable condition. Optimal disease management requires a multi-disciplinary, multi-professional team [117] based in a specialist centre, closely liaising with community care providers. The mainstay of therapy is addressing the existing/impending complications and symptom management [118]. Once widely available on the market, Enzyme replacement therapy (ERT) as a disease modifying agent is anticipated to slow the progression of non-CNS manifestations of disease [118, 119].

### How is optimal care delivered for a patient with ASMD?

*Statement 16*: Patients with ASMD exhibit variably progressive multisystem disease and benefit from multidisciplinary and multi-professional follow up from physicians and allied health care professionals with experience in this condition. Wherever possible, patients identified with ASMD should be referred to a centre with expertise in the care of this condition.

- Strength of recommendation: 1
- Level of evidence: A
- Experts’ opinion: completely agree (75%), mostly agree (19%), partially agree (6%), mostly disagree (0%) and completely disagree (0%).

Experience from other ultra-rare, multisystemic diseases showed that patients who have access to a highly specialised clinical service reported high levels of satisfaction in their care. Patient treatment compliance and clinic attendance was better in a multi-disciplinary clinic compared to the usual standard of care [120]. Depending on the country’s health care service setup, level of expertise and patient needs, a multi-disciplinary team (MDT) can be formed to enable ASMD patients to receive a collaborative management plan from a wide range of experts in an integrated manner. Specialists in the different disciplines (Table 4) have to work together to integrate information and care as much as possible.

**Table 4:** Recommended multidisciplinary assessment of patients with ASMD

Discipline	Features of ASMD for which this discipline may be of assistance	Recommended for all ASMD or as needed
Primary care physician	Assist with general medical care; coordinate specialists; provide support for family	All
Metabolic diseases specialist	Diagnosis of ASMD and exclusion of other disorders in the differential diagnosis; Ongoing patient assessment for disease progression and response to therapy. Coordinate the overall care working with primary care physician	All
Neurologist	Assess the possible neurological manifestation of the disease and manage accordingly	All
Hepatologist	Periodic assessments of liver derangements; Manage the impending/existing liver failure	As needed
Haematologist	Assess the risk of bleeding disorder and long term complications	As needed
Pulmonologist	Assess the baseline respiratory functions and periodic assessment for deterioration; manage the pulmonary disease and its complications	As needed
Genetic counsellor	To inform affected persons and their families regarding nature and implications of ASMD to facilitate medical and personal decision making; provide counselling for families as to recurrence risk and options for prenatal diagnosis if desired	All
Lipidologist/cardiologist	Manage the mixed dyslipidemia, and perform cardiovascular risk assessment for indicated primary or secondary prevention interventions	As needed
Psychiatrist/clinical psychologist	Assess for behavioural disturbances, depression and manage accordingly	As needed
Speech and language therapist	Assess for dysphagia and aspiration risk; Speech and feeding therapy for children with neuronopathic phenotypes	As needed
Occupational and physical therapists/rehabilitation physician	Assess and develop aids and home adjustments as needed for patients with communication and physical challenges	As needed
Nutritionist	Periodic assessments of nutritional status in patients who may be losing weight due to dysphagia or side effects of therapy; gastrostomy tube insertion as indicated	As needed
Social worker	Support of patients and families living with disabilities who require enhanced resources in the community	As needed
Developmental and behavioural paediatrician	Assess for the presence or absence of developmental delays in children; recommend appropriate therapies and educational interventions	As needed

International expert guidelines have been established here to monitor ASMD given the multi-systemic involvement and progressive nature of the disorder. Monitoring goals should be established at diagnosis and reviewed regularly, aimed at identifying and managing disease complications, and enhancing quality of life [119].

### How should burden of illness be assessed?

The clinical phenotype and life expectancy of patients with ASMD vary widely depending on the spectrum/type of the disease, age of onset, extents of target organ involvements and pre-existing/impending complications [47]. ASMD type A is the most severe form with a relatively homogenous natural history of rapid progression and short life expectancy [47] [38]. On the other hand, individuals with ASMD type B have a wide range of disease manifestations, variable rate of disease progression, severity level and life expectancy [30–32]. Individuals with ASMD type A/B have a phenotype intermediate between types A and B that typically includes a more slowly progressive neurodegenerative course. Recommendations for clinical monitoring of patients with ASMD have been published [119]. The following assessments should take place at the time of diagnosis or symptom onset and at regular intervals for optimal symptom control and maintain functional capacity (Table 5).

**Table 5:** Recommended assessments

Recommended assessment	Rationale	Frequency	Recommended for all ASMD or as needed
Baseline history	Establish natural history, systemic involvement, current level of disease severity and estimate rate of progression	At diagnosis	All
Interval history	Establish rate of disease progression; monitor for compliance with and side effects from therapy	3–12 monthly/each visit	As needed
Physical examination	Document growth parameters, assess for neurological features and organomegaly, assess for fatigue, abdominal pain, and/or bleeding tendency at least annually	At diagnosis then 6–12 monthly/each visit	As needed
Nutrition	Evaluation of nutritional status and safety of oral intake	At diagnosis then at each visit	As needed
Pulmonary assessment	Assess recurrent chest infections	At diagnosis then at each visit	All
	Assess for shortness of breath		
	Pulmonary function testing including assessment of diffusing capacity in persons old enough to cooperate	At diagnosis then annually	As needed
	Chest radiograph and/ or high resolution chest CT to assess extent of interstitial lung disease	At diagnosis regardless of age then every 2–4 years	All
Musculoskeletal assessment	Assess for fractures and/or extremity pain	At diagnosis then each visit	All
Neurologic assessment	Comprehensive neurologic evaluation, assess neurologic function and frequency of headaches	At diagnosis then annually	As needed
Ophthalmology evaluation	Presence of cherry-red spots at baseline and document	At diagnosis	All
Cardiac assessment (adult only)	EKG, echocardiogram, coronary angiogram as indicated	At diagnosis	As needed
		Every 3–5 years	
Blood investigations	Serum chemistries including liver transaminases (ALT, AST), albumin, and clotting factors to evaluate for progression of hepatic dysfunction	At diagnosis then at least annually	As needed
	Complete blood count to evaluate for thrombocytopenia, leukopenia, anemia, and increased bleeding		
	Measurement of lipid profile		
Imaging studies	Radiologic measurements of liver and spleen size as needed	At diagnosis then as needed	As needed
	Liver elastography or FibroScan to evaluate for hepatic fibrosis and cirrhosis		
Swallowing assessment	Swallowing assessment in all patients at risk; document presence of dysphagia and aspiration and response to therapy	At diagnosis and then 6 monthly in children; in adults, frequency could be reduced to every	As needed
		12 months if asymptomatic and disease is stable	
Developmental or cognitive assessment	Developmental assessment, monitor developmental progress and educational needs (evaluation for early intervention/special education)	At diagnosis then at each visit	As needed
	Document baseline degree of cognitive impairment including motor, adaptive, cognitive and speech/language and monitor response to therapy	At diagnosis; 6 monthly in children; 12 monthly in adults	As needed
Neuropsychiatric evaluation	Document psychiatric manifestations and response to therapy	At diagnosis then 6–12 monthly as indicated	As needed
Family support and resources	Assess need for family support and resources at each visit	At diagnosis then each visit	As needed
	Assess need for community or online resources such as Parent to parent; social work involvement for parental support		
	Home nursing referral		
	Assess for any change in social, domestic, or school or work related activities		

#### Growth and nutrition

*Statement 17*: The growth of children with ASMD (height, weight and head circumference) should be assessed at regular intervals as part of routine health assessments. In addition, adult patients should undergo a careful assessment of their anthropometric measurements.

- Strength of recommendation: 1
- Level of evidence: A
- Experts’ opinion: completely agree (69%), mostly agree (25%), partially agree (6%), mostly disagree (0%) and completely disagree (0%).

#### Developmental assessments

*Statement 18*: Children with ASMD should have assessments of their age-appropriate acquisition of developmental milestones. Developmental screens can be performed by primary health care providers, and more formal age-appropriate developmental assessments should be performed as part of MDT assessments. Those with developmental milestones concern should have accesses to early intervention and development support.

- Strength of recommendation: 1
- Level of evidence: A
- Experts’ opinion: completely agree (87%), mostly agree (13%), partially agree (0%), mostly disagree (0%) and completely disagree (0%).

Delayed acquisition of developmental milestones is seen in patients with ASMD type A and common in children with type A/B. Regular evaluation of their motor and cognitive function, speech and language is indicated. Consideration should be given to changes in these abilities that may impact on daily living activities. Testing should be age and functionally appropriate, using standardised assessment tools. Strategies to ensure the safety of the patient’s environment and the availability of support mechanisms are essential to improve the quality of life of the patient/families. Appropriate ongoing education and developmental support into adulthood and beyond is required.

#### Physical examination

*Statement 19*: Individuals with ASMD should undergo a comprehensive physical examination including detailed neurological assessment at the time of diagnosis and thereafter on regular interval.

- Strength of recommendation: 1
- Level of evidence: A
- Experts’ opinion: completely agree (81%), mostly agree (6%), partially agree (13%), mostly disagree (0%) and completely disagree (0%).

Detailed neurologic examination is particularly important for newly diagnosed children, especially when the ASMD type has not yet been established. The presence or absence of neurologic findings may help determine the phenotype and enable more accurate prognostics.

#### Routine monitoring laboratory tests

*Statement 20*: Biochemical and haematological abnormalities are common in patients with ASMD and hence they need blood tests at baseline and regular interval. These include but are not limited to, full blood cell count, liver enzymes, vitamin D, lipid profile, clotting markers, enhanced liver fibrosis test, lysosphingomyelin and PPCS.

- Strength of recommendation: 1
- Level of evidence: B
- Experts’ opinion: completely agree (44%), mostly agree (44%), partially agree (12%), mostly disagree (0%) and completely disagree (0%).

Haematological abnormalities such as thrombocytopenia, leukopenia and anemia are common ranging from 21 to 53% study population [41]. Mixed dyslipidaemia with atherogenic lipoprotein profile is highly suggestive of ASMD. Liver function test abnormalities such as raised transaminases can occur in up to 75% of patients with ASMD [31, 38]. Lysosphingomyelin appears to be a valuable biomarker for overall ASMD disease severity [84].

#### Evaluation of liver and spleen

*Statement 21*: Hepatosplenomegaly is present in most ASMD patients at the time of diagnosis. We recommend liver and spleen MRI including volumetric assessment, although ultrasounds can be performed in younger patients and where resource is limited.

- Strength of recommendation: 2
- Level of evidence: B
- Experts’ opinion: completely agree (69%), mostly agree (31%), partially agree (0%), mostly disagree (0%) and completely disagree (0%).

The most common observation at presentation is splenomegaly (78%) and hepatomegaly (73%) [41]. The sphingomyelin deposition in the liver poses an increased risk of progression to fibrosis, cirrhosis and eventually liver failure and its related complications. Liver failure is the second common cause of death in patients with ASMD of type B [43]. Non-invasive evaluation of liver fibrosis by ultrasound/MRI and elastographic techniques at regular interval is warranted [119].

#### Evaluation of pulmonary disease

*Statement 22*: Interstitial lung disease occurs in the majority of ASMD patients at some time in their lives, especially in the younger ones. Chest X ray and high resolution chest CT scan should be performed at baseline and regular interval as required. Impaired O_2_/CO_2_ exchange is reflected by compromised diffusion capacity. This may be associated with shortness of breath and greater disease severity. Chest tomography is the most useful imaging modality to evaluate the interstitial lung disease, with typical ground glass appearance. Pulmonary function tests (especially DLCO –diffusion capacity for carbon monoxide) and exercise testing are important to detect impaired diffusion capacity.

- Strength of recommendation: 1
- Level of evidence: B
- Experts’ opinion: completely agree (81%), mostly agree (19%), partially agree (0%), mostly disagree (0%) and completely disagree (0%).

Pulmonary pathology in ASMD typically manifests as interstitial lung disease (ILD) caused by sphingomyelin accumulation in alveolar and intra-alveolar macrophages within the alveolar septum. This results in distortion and thickening of the alveolar septum, and impaired O_2_/CO_2_ exchange [121].

Pulmonary dysfunction is a key clinical characteristic of all ASMD phenotypes. Pulmonary involvement affects many patients with ASMD to some degree, with some patients experiencing progressive lung deterioration and respiratory failure [38, 119, 122]. However, several attenuated adult patients may have no evidence of lung disease [30].

For patients with ASMD type A, pulmonary manifestations can include frequent infections (e.g., pneumonia) and respiratory arrest [29]. For patients with ASMD type B and A/B, pulmonary manifestations can include: frequent infections, shortness of breath, and exercise dyspnea and reduced exercise tolerance [38, 41, 47, 122]. Diffusing capacity of carbon monoxide (DLCO) is a clinically meaningful measure of disease burden for patients with ASMD [122]. However, due to the rarity of ASMD and often inadequate diagnostic screening initiatives, available evidence remains limited, especially on mortality/survival, frequency, and timing of significant clinical events [43, 122].

#### Evaluation of cardiovascular disease

*Statement 23*: Adult patients with ASMD usually have an atherogenic lipid profile and hence may be at an increased risk of premature cardiovascular events with age. Appropriate assessments should be performed as clinically indicated.

- Strength of recommendation: 2
- Level of evidence: C
- Experts’ opinion: completely agree (56%), mostly agree (31%), partially agree (13%), mostly disagree (0%) and completely disagree (0%).

Dyslipidemia with low high-density lipoprotein cholesterol, increased low density lipoprotein cholesterol, and hypertriglyceridemia appears to be associated with early atherosclerotic heart disease [46] in line with the general population. To date, increased risk of premature CV events is not proven despite atherogenic lipid profile as well as coronary artery status. Therefore, the use of lipid lowering therapy (e.g. statins) in ASMD patients as a primary prevention needs careful consideration in the context of the overall cardiovascular risk of the individual.

#### Evaluation of skeletal disease

*Statement 24*: Individuals with ASMD may be at risk of osteopenia and osteoporosis. Bone density studies could be performed in individuals as clinically indicated.

- Strength of recommendation: 2
- Level of evidence: C
- Experts’ opinion: completely agree (63%), mostly agree (31%), partially agree (6%), mostly disagree (0%) and completely disagree (0%).

Adults with ASMD type B may have some degree of osteopenia or osteoporosis. Children with ASMD may also have low Z scores for bone mineral content and density [45]. Pathologic fractures have been reported in some ASMD individuals with severe disease [123].

### Disease modifying therapy

**Olipudase alfa**: is an enzyme replacement therapy addressing the underlying metabolic defect by replacing deficient or defective acid sphingomyelinase. To date, it is the first and only disease modifying therapy for patents with ASMD.

*Statement 25*: Olipudase alfa, an enzyme replacement therapy (ERT) using human recombinant acid sphingomyelinase, is indicated as a disease-modifying enzyme replacement therapy for the long-term treatment of non-central nervous system (CNS) manifestations of ASMD. At the time of writing, olipudase alfa has received regulatory approval in Brazil, Japan, Europe and the United States of America and is awaiting approval in other countries.

- Strength of recommendation: 1
- Level of evidence: A
- Experts’ opinion: completely agree (69%), mostly agree (31%), partially agree (0%), mostly disagree (0%) and completely disagree (0%).

*Statement 26*: All patients with a confirmed diagnosis of ASMD and significant non-Central Nervous System (CNS) manifestations could be considered for olipudase alfa therapy on an individual basis.

- Strength of recommendation: 1
- Level of evidence: A
- Experts’ opinion: completely agree (65%), mostly agree (29%), partially agree (6%), mostly disagree (0%) and completely disagree (0%).

*Statement 27*: The effectiveness of treatment with olipudase alfa should be monitored with the measurement of growth (in children), the volume of liver and spleen, lung function, haematological markers, plasma lipid profile and disease biomarkers and data should be collected for future treatment guideline development.

- Strength of recommendation: 1
- Level of evidence: A
- Experts’ opinion: completely agree (94%), mostly agree (6%), partially agree (0%), mostly disagree (0%) and completely disagree (0%).

Four studies of olipudase alfa have been completed. A single-center, open-label, single-ascending-dose trial evaluated the safety of olipudase alfa (0.03–1.0 mg/kg) in 11 adults with ASMD type B (NCT00410566). This study identified 0.6 mg/kg as a maximum tolerated starting dose that supported a dose-escalation strategy [124]. A Phase 1B study assessed safety and tolerability in five adult patients with non neuronopathic ASMD that received escalating doses of olipudase alfa every 2 weeks for 26 weeks (NCT01722526) [125, 126].

A Phase II/III, international multicentre, randomized, double-blind, placebo-controlled trial (ASCEND; NCT02004691/EudraCT 2015-000371-26) enrolled 36 adults with ASMD randomized 1:1 to receive olipudase alfa or placebo intravenously every 2 weeks with intrapatient dose escalation to 3 mg/kg. Primary efficacy endpoints were percent change from baseline to week 52 in percent predicted diffusing capacity of the lung for carbon monoxide and spleen volume. Least square mean percent change from baseline to week 52 favoured olipudase alfa over placebo for percent predicted diffusing capacity of the lung for carbon monoxide (22% vs. 3.0% increases, *P* = 0.0004), spleen volume (39% decrease vs 0.5% increase, *P* < 0.0001), and liver volume (28% vs. 1.5% decreases, *P* < 0.0001) [86].

In addition, (ASCEND-Peds/NCT02292654) study was designed as phase I/II, international, multicenter, open-label trial that enrolled 20 paediatrics patients to receive intravenous olipudase alfa every 2 weeks with intrapatient dose escalation to 3 mg/kg. Primary outcome was safety through week 64. In this study in children with ASMD, olipudase alfa was generally well-tolerated with significant improvements in disease pathology across a range of clinically relevant endpoints [85]. The most commonly observed adverse reactions included headache, cough, diarrhoea, and hypotension in adults, and pyrexia, cough, diarrhoea, rhinitis, and abdominal pain in children. Importantly, hypersensitivity reactions including anaphylaxis occurred in some paediatric patients. In summary, olipudase alfa was well-tolerated in children and adults and, after treatment (1 year in adult and 2 years in children), resulted in improved lung function, reductions in spleen and liver volumes, improved platelet counts and lipid profiles, reductions in disease biomarkers, and improved growth (in children) [85, 86, 127–129].

The guideline development group had extensive discussion about olipudase alfa use in patients with acute or rapidly progressive neurologic disease, as they were excluded from the ASCEND-Peds study (NCT02292654/Sanofi Genzyme) given the inability of the intravenously administered enzyme to cross the blood brain barrier or impact neurologic disease. However, early managed access programs allowed some patients with visceral and neurologic manifestations of ASMD access to olipudase alfa with a focus on ameliorating visceral signs and symptoms [130, 131]. National patient organizations surveyed parents of children who received olipudase alfa for at least 12 months, including some patients with visceral and neurologic manifestations of ASMD. Data from that survey suggests all parents were satisfied with the therapy and perceived a meaningful benefit in controlling visceral symptoms (submitted manuscript).

Some countries have more liberal indications that allow for olipudase alfa use in all ASMD patients, including infants with acute neurovisceral and rapidly progressive chronic neurovisceral ASMD. As olipudase alfa will not prevent or impact CNS manifestation, it is important that families of infants with rapidly progressive neuronopathic forms of ASMD who are considering olipudase alfa are counselled about the risks, benefits, limitations, and long-term futility of therapy, as well as the option to terminate treatment when progressive neurodegeneration occurs. Alternatives to disease modifying therapy, supportive management, family counselling, and palliative care, should also be discussed.

Patients eligible for ERT should undergo disease burden assessment prior to treatment initiation in order to assess the potential benefit of olipudase alfa on the respiratory function, spleen and liver volume, platelet count, growth in children and quality of life measures. Individually tailored treatment goal is agreed between the care givers and patients/families at the outset, and these assessments should be repeated on regular interval. In addition, other issues related to tolerance, treatment adherence and comorbidities should be monitored.

#### Haematopoietic stem cell transplantation

*Statement 28*: Variable results have been reported with haematopoietic stem cell transplantation (HSCT) and morbidity such as graft versus host disease (GvHD), infection and renal tubular dysfunction, and mortality associated with HSCT limits its use. HSCT may be useful to correct the metabolic defect, improve blood counts, and reduce increased liver and spleen volumes, but does not address neurologic disease and the revers of growth retardation is uncertain. Therefore, any attempts to perform HSCT in individuals with clinically evident neurologic disease should be considered experimental as it does not correct or stabilize neurologic disease.

- Strength of recommendation: 2
- Level of evidence: C
- Experts’ opinion: completely agree (56%), mostly agree (31%), partially agree (13%), mostly disagree (0%) and completely disagree (0%).

### Symptoms management

#### What optimal symptomatic therapy should be considered for a patient with ASMD deficiency?

##### Liver disease and splenomegaly

*Statement 29*: Liver enlargement with elevated transaminases is common in ASMD, which may progress to fibrosis and cirrhosis in the third to fourth decade. In some severely affected individuals, liver failure may occur resulting in portal hypertension with associated oesophageal varices, ascites, and hepatic encephalopathy. Close monitoring of liver function and early consultation with a hepatologist is recommended as needed. Splenectomy should be avoided.

- Strength of recommendation: 2
- Level of evidence: C
- Experts’ opinion: completely agree (81%), mostly agree (19%), partially agree (0%), mostly disagree (0%) and completely disagree (0%).

Advanced cases of chronic liver disease (CLD) with fulminant liver failure were reported in a few adults with ASMD [38, 42]. The rate of progression of liver disease is extremely variable [31, 41, 43]. More research is needed to determine the cause and rate of progression of advanced CLD in ASMD patients. Liver failure is one of a leading cause of mortality in ASMD patients [38] and adult patients with evidence of transaminitis/fibrosis should be followed by hepatologists. Liver biopsy in persons with evidence of deteriorating liver function may be indicated if non-invasive means to ascertain fibrosis are not available [119]. If patients are known to have advanced CLD then they should be monitored and treated for risk of gastrointestinal bleeding and surveillance for hepatocellular cancer. The outcomes of liver transplantation have been reported in several individuals with ASMD type B who had progressive liver dysfunction. In addition to improvements in hepatic function and dyslipidemia, significant improvements in lung disease and paediatric growth parameters were observed [41, 119, 132, 133].

Splenectomy on the other hand is not recommended as it may lead to exacerbation of liver disease and increased sphingomyelin accumulation in the lungs causing progressive respiratory insufficiency. If splenectomy is required due to massive splenomegaly, pressure symptoms, and severe unsustainable hypersplenism, then partial splenectomy or partial splenic arterial embolization are options. If partial or total splenectomy should be performed according to surgical indications, standard post-surgical antibiotic prophylaxis and vaccinations should be used.

##### Respiratory system

*Statement 30*: ASMD patients with pulmonary and/or neurological disease are at an increased risk of frequent respiratory infection including aspiration pneumonia and care givers should be vigilant in preventing and/ or promptly managing respiratory infection. Older individuals with ASMD should have their history reviewed for respiratory symptoms and lung function test along with the need for non-invasive ventilation. Vaccination against influenza, COVID-19 and Streptococcus pneumoniae should be encouraged.

- Strength of recommendation: 2
- Level of evidence: C
- Experts’ opinion: completely agree (63%), mostly agree (25%), partially agree (12%), mostly disagree (0%) and completely disagree (0%).

Respiratory disease is a primary and independent contributor to mortality in ASMD type A (27.7% of cases) [43], and disease burden and morbidity for patients with chronic forms of ASMD [31, 33, 41, 42, 47]. Progressive interstitial lung disease is a prevalent clinical feature of ASMD contributing to decreased QoL and increased disease burden.

Patients with progressive pulmonary disease may require long-term oxygen therapy. Other treatment for interstitial lung disease (e.g., steroids) and therapeutic lung lavage may be indicated. Endoscopic treatment of bronchial casts may be urgently required. Lung transplantation does not have proven extra benefit. Supporting measures such as smoking cessation should be encouraged. Other treatments for interstitial lung disease (e.g., steroids) have not been well studied [134, 135]. Therapeutic bronchopulmonary lavage for some patients with ASMD type-B has been associated with temporary clinical improvement, but with variable results [117, 134, 135]. Four cases of lung transplantation in ASMD type-B with variable results have been reported [136–139].

##### Haematology

*Statement 31*: Bleeding tendency is common but the type and severity of bleeding is highly variable. Consultation with a haematologist in case of severe thrombocytopenia is recommended for evaluation and management.

- Strength of recommendation: 2
- Level of evidence: C
- Experts’ opinion: completely agree (69%), mostly agree (31%), partially agree (0%), mostly disagree (0%) and completely disagree (0%).

The most common bleeding event is epistaxis and mostly self-limiting. However, significant bleeding event such as post-surgical and trauma-related haemorrhages and life-threatening, liver-disease associated oesophageal variceal disease can occur [38, 43]. Although thrombocytopenia is a common manifestation of ASMD, the platelets are rarely low enough to cause significant bleeding suggesting the presence of additional factors that impact clotting.

##### Swallowing and diet

*Statement 32*: Children with neuronopathic ASMD (types A and A/B) should undergo a comprehensive swallowing assessment by a speech and language therapist. Instruction in dietary modification and compensatory postures may be beneficial for individuals with dysphagia. The family should be educated regarding the progressive worsening of swallowing skills and increased risk of aspiration as part of an ongoing care. Nasogastric tube feeding or surgical placement of feeding tube can be considered to enhance caloric intake and possibly reduce the risk of aspiration, although the family should be counselled that this is optional given that ASMD type A is, at present, uniformly fatal.

- Strength of recommendation: 2
- Level of evidence: B
- Experts’ opinion: completely agree (69%), mostly agree (31%), partially agree (0%), mostly disagree (0%) and completely disagree (0%).

##### Irritability and sleep disturbance

*Statement 33*: Clinicians and caregivers of individuals with ASMD Type A should be aware that there is an increased prevalence of irritability and sleep disturbance affecting quality of life for entire family. Management plans for irritability and sleep disturbance should be considered as indicated.

- Strength of recommendation: 2
- Level of evidence: C
- Experts’ opinion: completely agree (69%), mostly agree (31%), partially agree (0%), mostly disagree (0%) and completely disagree (0%).

##### Chronic pain and fatigue

*Statement 34*: Chronic pain and fatigue may occur in the majority of ASMD patients at some time in their lives. Optimising pain management and assessment for fatigue should be performed as indicated according to the local standards.

- Strength of recommendation: 2
- Level of evidence: C
- Experts’ opinion: completely agree (50%), mostly agree (44%), partially agree (6%), mostly disagree (0%) and completely disagree (0%).

##### Psychosocial wellbeing

*Statement 35*: Clinicians, caregivers and individuals with ASMD should be aware that there is an increased prevalence of behavioural problems and other psychiatric disorders such as anxiety and depression in ASMD. There should be a low threshold for referral to a clinical psychology/psychiatric team as appropriate, and for the use of both non-pharmacological and/or pharmacological treatments.

- Strength of recommendation: 1
- Level of evidence: B
- Experts’ opinion: completely agree (62%), mostly agree (19%), partially agree (0%), mostly disagree (19%) and completely disagree (0%).

As with most ultra-rare diseases, there is a limited robust data regarding the disease burden and the impact on the psychosocial wellbeing of an individual with ASMD and their immediate family members [49]. In a small study based on interviews with patients and caregivers, numerous psychosocial stressors such as social isolation, peer rejection, chronic pain, fatigue and living with a life-threatening disease were associated with high level of stress, anxiety and depression [48]. In addition, children with ASMD type A show increased signs of irritability, prolonged crying and sleep disturbance [29]. Similarly, depression and psychosis requiring anti-depressant/psychotic therapies may occur in adult patients with ASMD [47].

### Transition, family and reproductive care and advanced care planning

#### Transition

*Statement 36*: Most children with chronic visceral ASMD are expected to reach adulthood with complex medical and psychosocial needs. The process of transition from paediatric to adult services should begin early and gradually progress allowing patients and their caregivers to build a relationship and develop confidence with their adult metabolic team. Therefore, the clear communication and trust between these two teams would be highly beneficial for patients to feel continuously cared for at the highest standards by their new team. It must also include appropriate services in the community to provide a seamless transition from childhood to adult life. Individuals with ASMD may benefit from a detailed assessment identifying barriers to independence.

- Strength of recommendation: 1
- Level of evidence: A
- Experts’ opinion: completely agree (69%), mostly agree (31%), partially agree (0%), mostly disagree (0%) and completely disagree (0%).

#### Family and reproductive care

*Statement 37*: Once the SMPD1 pathogenic variants have been identified in an affected family member, diagnostic testing of all at-risk family members is warranted to allow for early diagnosis and treatment of ASMD. All patients identified pre-symptomatically should be referred to specialist centres for surveillance and early detection of disease progression.

- Strength of recommendation: 1
- Level of evidence: A
- Experts’ opinion: completely agree (75%), mostly agree (25%), partially agree (0%), mostly disagree (0%) and completely disagree (0%).

Molecular genetic testing is recommended for the parents of a proband to confirm that both parents are heterozygous for an *SMPD1* pathogenic variant and to allow testing for at risk relatives. Either test for the familial *SMPD1* pathogenic variants or measure residual acid sphingomyelinase enzyme activity is appropriate to detect affected individuals.

Individuals at risk will require careful genetic counselling by genetics professionals to inform affected persons and their families regarding nature and implications of ASMD to facilitate medical and personal decision making.

*Statement 38*: Prenatal ASMD testing for a pregnancy at increased risk should be offered to all at risk couples, subject to local protocols and laws. Molecular testing for the familial SMPD1 variants using chorionic villus sampling (CVS) or amniotic fluid sampling is the most common means of testing at risk pregnancies. Biochemical prenatal diagnosis by testing of ASM enzyme activity in CVS or cultured amniocytes may also be used for at risk pregnancies.

- Strength of recommendation: 2
- Level of evidence: B
- Experts’ opinion: completely agree (63%), mostly agree (31%), partially agree (6%), mostly disagree (0%) and completely disagree (0%).

The determination of genetic risk and discussion of the availability of prenatal/preimplantation genetic testing should be carried out before pregnancy. Genetic counselling (including discussion of potential risks to offspring and reproductive options) should be offered to young adults who are affected, are carriers, or are at risk of being carriers.

#### Advance care planning

*Statement 39*: Specialist centre care providers, family physician/ paediatrician, health care decision makers, local palliative care services and patient advocates should develop close working links to select treatments and develop disease management plans that address patients’ holistic needs in order to support individuals, caregivers and families with ASMD through the lifespan, including: (a) advance care planning with regular updating, (b) proper flow of communication and information for patients and their families, and (c) a designated point of contact for each stage in their care pathway. An individual identified as being near the end of life may benefit from ongoing access to palliative care services including for symptom control, respite, psychological and spiritual support.

- Strength of recommendation: 1
- Level of evidence: A
- Experts’ opinion: completely agree (81%), mostly agree (19%), partially agree (0%), mostly disagree (0%) and completely disagree (0%).

## Conclusion

These guidelines are the result of an international collaboration of experts in the care of ASMD and the evidence gathered to write the guidelines are the best evidence available to the experts. The guidelines address the management of patients affected by subtypes of ASMD and are intended to facilitate optimal care to all ASMD patients regardless of their demography and access to health care. In addition, it defines the standard of care against which practice can be audited and best practices can be disseminated. The Guidelines Development Group commits itself to revise this work in 3–5 years’ time to reflect new data pertaining to future research findings and new therapies.

## Data Availability

Data sharing not applicable to this article as no datasets were generated or analysed during the current study.

## Abbreviations

7KC: 7-Ketocholesterol
AGREE II: Appraisal of Guidelines for Research and Evaluation
ASMD: Acid sphingomyelinase deficiency
BMC: Bone mineral content
BMD: Bone mineral density
C-triol: Cholestane-3β,5α,6β-triol
CHQ-PF50: Child Health Questionnaire-Parental Form 50 for paediatric patients
CLD: Chronic liver disease
CNV: Copy number variations
DBS: Dried blood spots
DLCO: Diffusing capacity of carbon monoxide
ERT: Enzyme replacement therapy
GDG: Guidelines development group
GRADE: Grading of recommendations, assessment, development and evaluations
HSCT: Haematopoietic stem cell transplantation
INPDA: International Niemann–Pick disease alliance
INPDR: International Niemann–Pick disease registry
LAL-D: Lysosomal acid lipase deficiency
LSD: Lysosomal storage diseases
lyso-Gb1: Glucosylsphingosine
lyso-SM: Lysosphingomyelin
lyso-SM509: Lysosphingomyelin-509
MDT: Multidisciplinary team
MoM: Multiple of median of controls
NPC: Niemann–Pick disease type C
NPD: Niemann–Pick disease
PPCS: *N*-palmitoyl-*O*-phosphocholine-serine (previously named lyso-SM509
QoL: Quality of life
RCT: Randomized controlled trials
SF-36: Short-Form 36
SPC: Sphingosylphosphorylcholine
TCG: N-(3β,5α,6β-trihydroxycholan-24-oyl) glycine
VUS: Variants of uncertain significance
